# Machine learning-based global maps of ecological variables and the challenge of assessing them

**DOI:** 10.1038/s41467-022-29838-9

**Published:** 2022-04-22

**Authors:** Hanna Meyer, Edzer Pebesma

**Affiliations:** 1 Institute of Landscape Ecology, Westfälische Wilhelms-Universität Münster, Heisenbergstraße 2, Münster, 48149 Germany; 2 Institute for Geoinformatics, Westfälische Wilhelms-Universität Münster, Heisenbergstraße 2, Münster, 48149 Germany

**Keywords:** Conservation biology, Ecological modelling, Ecological modelling

## Abstract

The recent wave of published global maps of ecological variables has caused as much excitement as it has received criticism. Here we look into the data and methods mostly used for creating these maps, and discuss whether the quality of predicted values can be assessed, globally and locally.

Fields such as ecology or geosciences have seen a strong increase of studies that apply machine learning methods to produce global maps of environmental variables (prominent examples are, e.g., the global tree restoration potential^1^, global soil nematode abundances^2^, or global soil maps^3^) with the aim of increasing our knowledge about the environment, and of supporting decisions. These maps are often distributed as open data, allowing other researchers to use them as input to compute indicators of all kinds or as input to map yet other variables. Quality measures reported by the authors are impressive but often contradict with experts’ opinions (e.g., see comments to Bastin et al.^1^ or discussions in Wyborn and Evens^4^). Ploton et al.^5^ attribute this contradiction to the use of validation strategies that ignore spatial autocorrelation in the data, and argue in favor of using spatial cross-validation methods. Wadoux et al.^6^ argue that spatial cross-validation is not the right way to evaluate map accuracy. Meyer and Pebesma^7^ argue that the practice of using sparse and non-representative reference data makes model assessment impossible for areas with conditions that are very different from the training data. Here, we try to unravel some of these arguments by focusing on the data, the methods used, and the limits to our ability to assess spatial predictions.

## Global reference data used in machine learning applications

In common global predictive mapping tasks (described in, e.g., Van den Hoogen et al.^8^), models are trained using reference data from field sampling. These data are then spatially matched with predictor variables with global coverage. A machine learning model (often Random Forest) is then fitted (trained) and applied to the predictors to obtain a global map with predicted values of the target variable.

Most machine learning methods as well as common validation strategies assume that the reference data are independent and identically distributed, which is in the spatial mapping context for instance guaranteed when they were obtained as a simple random sample from the target area. It is, however, hard to imagine that a global, spatially random sample will ever be collected when it involves taking in situ samples (e.g., collecting soil parameters, or counting soil nematodes). None of the global studies mentioned above is based on data collected as a probability sample; most of them are based on creating a database by merging all data available from different sources. As a consequence, these data are strongly concentrated, e.g., in Europe and Northern America, and within these regions, they are extremely clustered around areas that received intense research. We are aware that large gaps in geographic space do not always imply large gaps in feature space, but it is the former that most concerns accuracy of the maps of focus here, as we will discuss.

**Fig. 1:**
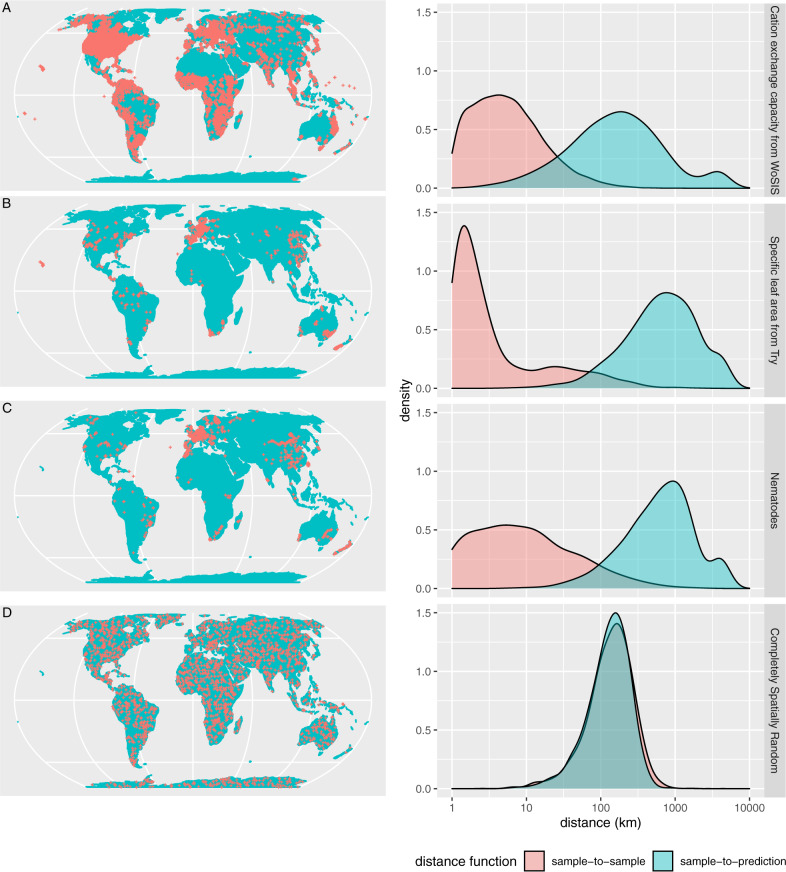
Spatial distance distributions in global mapping studies. Spatial distribution (left; equal Earth projection) and distribution of nearest neighbor distances (right; sample-to-sample distance in pink, prediction-location-to-sample distance in blue) for three different publicly available datasets: cation exchange capacity in the soil from the WoSIS database^23^ as used for global soil mapping^3^ (**A**), specific leaf area from the Try database^24^ as used for the global mapping in Moreno-Martinez et al. (2018)^25^ (**B**), and the nematodes dataset compiled by Van den Hoogen et al. (2019)^2^ (**C**). For comparison, the fourth dataset is a simulated completely spatially random sample of the same size as the nematode dataset (**D**). Distance distributions were calculated and visualized using the R package “CAST”^26^.

For three publicly available datasets that were used for global mapping, Fig. 1A–C compares the distributions of the spatial distances of reference data to their nearest neighbor (pink) with the distribution of distances from all points of the global land surface to the nearest reference data point (prediction locations, blue). The difference between the two distributions reflects the degree of spatial clustering in the reference data: Fig. 1D shows the distributions for a simulated spatially random sample of the same size as Fig. 1C. The clustered pattern has certain consequences and raises challenges for accuracy assessment that we will discuss in the following.

## Map quality: global or local assessment?

The quality of global maps can be assessed in different ways. One way is global assessment where a single statistic is chosen to summarize the quality of the entire map: the map accuracy. For a categorical variable, this can be the probability that for a randomly chosen location on the map, the map value corresponds to the true value. For a continuous variable, it can be the RMSE, describing for a randomly chosen location on the map the expected difference between the mapped value and the true value. When a probability sample, such as a completely spatially random sample, is available for the area for which a global assessment is needed, then map accuracy can be estimated model-free (also called design-based, e.g., by using the unweighted sample mean in case of a completely spatially random sample). This circumvents modeling of spatial correlation because observations are independent by design^6,9^. This approach is called model-free because no model needs to be assumed about the distribution or correlation of the data: the only source of randomness is the random selection of sample units from a target population. If a probability sample is not available this approach cannot be used, and automatically the accuracy assessment approach becomes model-based^10^, which involves modeling a spatial process by assuming distributions and taking spatial correlations into account, and choosing estimation methods accordingly.

Using naive random *n*-fold or leave-one-out cross-validation methods (or a simple random train-test split) to assess global model quality (usually equated with map accuracy) makes sense when the data are independent and identically distributed. When this is not the case, dependencies between nearby samples, e.g., in a spatial cluster, are ignored and result in biased, overly optimistic model assessment, as shown in, e.g., Ploton et al.^5^. Alternative cross-validation approaches such as spatial cross-validation^5,11^ that control for such dependencies are the only way to overcome this bias. Different spatial cross-validation strategies have been developed in the past few years, all aiming at creating independence between cross-validation folds^5,11–13^. Cross-validation creates prediction situations artificially by leaving out data points and predicting their value from the remaining points. If the aim is to assess the accuracy of a global map, the prediction situations created need to resemble those encountered while predicting the global map from the reference data (see Fig. 1 and discussions in Milà et al.^14^). This occurs naturally when reference data were obtained by (completely spatially random) probability sampling, but in other cases, this has to be forced for instance by controlling spatial distances (spatial cross-validation). Such forcing, however, is only possible when the distances in space that need to be resembled are available in the reference data. In the extreme case where all reference data come from a single cluster, this is impossible. When all reference data come from a small number of clusters, larger distances are available between clusters but do not provide substantial independent information about variation associated with these distances. Lack of information about larger distances means that we cannot assess the quality of predictions associated with such distances and cannot properly estimate global quality measures. Alternative approaches such as experiments with synthetic data^15^ or a validation using independent data at a higher level of integration^16^ would then be options to support confidence in the predictions.

Another way of accuracy assessment is local assessment: for every location, a quality measure is reported, again as probability or prediction error. Such a local assessment predicts how close the map value is to newly observed values at particular locations. If the measurement error is quantified explicitly, a smoother, measurement-error-free value may be predicted^10^. If the model accounts for change of support^10,17^, predictions errors may refer to average values over larger areas such as 1 × 1, 5 × 5, or 10 × 10 km grid cells. Examples of local assessment in the context of global ecological mapping are modeled prediction errors using Quantile Regression Forests^18^ or mapped variance of predictions made by ensembles^1,2^. Neither of these examples quantifies spatial correlation or measurement error, or addresses change of support, as it is known from other modeling frameworks^19^. By omitting to model the spatial process, the local accuracy estimates as presented in the global studies that motivated this comment are disputable.

The difference between global and local assessment is striking, in particular for global maps. A global, single number averages out all variability in prediction errors, and obscures any differences, e.g., between continents or climate zones. It is of little value for interpreting the quality of the map for particular regions.

## Limits to accuracy assessment

Maps, and in particular global maps, create a strong feeling of satisfaction, suggesting we now know it all. They are however also used, enlarged, torn apart, read in detail, and may form the basis for local decisions of all kinds, or even form the inputs for follow-up models. If a global map does not come with clear instructions about its value, like a prescription for subsequent use, it is easy to abuse it. Wyborn and Evans^4^ rightly ask about “what changes are global maps, and their creators, trying to bring about in the world?”, and suggest a re-engagement with empirical studies of local and regional contexts while seeking co-construction with those having local knowledge. The fact that creating global maps of anything nowadays is so easy does not mean these maps are always useful.

Technically, a trained Random Forest (or other) model can be applied globally as long as global predictors are available. Predictions far beyond reference data, however, often lead to extrapolation situations in the predictor space and models produce typically meaningless predictions when provided with predictor values that do not resemble the training data. The same applies to local accuracy estimates when based on the variance of predictions^7^. A good coverage of training data in the predictor space is hence required to produce globally applicable predictions. Since distances in geographic space often go along with distances in the feature space, it can be assumed that this is not given for many prediction models that are based on sparse and clustered reference data. In Meyer and Pebesma^7^, we suggest a procedure to limit spatial predictions to the area of applicability of the model: global maps would need to gray out areas where predictor values are too different from values in the training data—the areas for which we cannot assess the quality of predictions. Similar approaches have been suggested and discussed, e.g., by Jung et al.^16^. Limiting predictions to the area of applicability of the model is not only relevant to avoid wrong conclusions about prediction patterns but also to avoid propagation of large errors: many global maps of environmental variables used the global soil maps produced by Hengl et al.^3^ as input predictors^1,2,20^. The global soil maps by Hengl et al.^3^ in turn used other modeled maps as an input (e.g., WorldClim^21^). If the latter maps had labeled locations with predictions for which quality cannot be assessed, or for which quality was really low, the follow-up study could have benefited from it. Without that information, both WorldClim and the soil layers were taken as if they contained true values.

We argue that showing predicted values on global maps without reliable indication of global and local prediction errors or the limits of the area of applicability, and distributing these for reuse, is not congruent with basic scientific integrity. Reusing such global maps while ignoring prediction errors amplifies this problem, hence more transparency and clear indication about the limitations of predictions is required. Global maps are being distributed digitally and could be used for purposes of decision making, e.g., in the context of nature conservation^22^. We call for global maps of ecological variables to be published only when they are accompanied by properly derived local and global accuracy measures.
